# Concomitant Presence of CD5-Positive Diffuse Large B-Cell Lymphoma and Monoclonal B Cells with the “CLL Immunophenotype” - Is It Richter’s Transformation?

**DOI:** 10.4274/tjh.2016.0451

**Published:** 2017-06-01

**Authors:** Sabina Langer, Jasmita Dass, Suchi Mittal, Shyam Aggarwal

**Affiliations:** 1 Sir Ganga Ram Hospital, Clinic of Hematology, New Delhi, India

**Keywords:** Richter’s syndrome, Flow cytometry, Chronic lymphocytic leukemia, CD5-positive, Diffuse large B-cell lymphoma

## To The Editor,

The presence of diffuse large B-cell lymphoma (DLBCL) with a concomitant unsuspected population of B cells with chronic lymphoid leukemia (CLL) phenotype is very rare with no antecedent history of CLL. This may represent cases of de novo Richter’s transformation or the coexistence of two neoplasms [1]. In cases where the monoclonal B-cell population does not exceed 5x10^9^/L, this may represent DLBCL with concomitant monoclonal B-cell lymphocytosis (MBL) of the CLL phenotype. The coexistence of MBL of the CLL phenotype creates a diagnostic conundrum, especially in an unusual case of CD5^+^ DLBCL as it may be a de novo CD5^+^ DLBCL or Richter transformation [2]. The former has an aggressive course compared to de novo DLBCLs [3,4]. CLL has been known to occur synchronously or metachronously with hairy cell leukemia [5] and DLBCL [6]. We present here a case of clinically aggressive de novo CD5^+^ DLBCL with an unsuspected second population of CLL-like MBL detected on flow cytometry.

**Figure 1:**
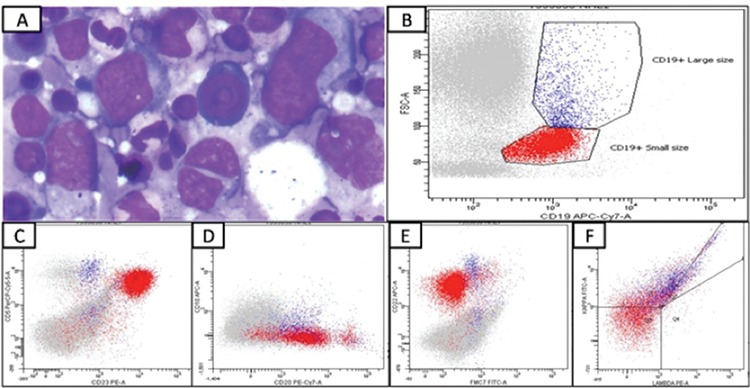
A) Bone marrow aspirate with 10%-15% abnormal lymphoid cells (3-5 times the size of a small mature lymphocyte with moderate amount of deep blue cytoplasm, round nuclei with irregularity of membranes in some, and coarsely clumped chromatin (Wright-Giemsa, 1000^x^). B) Flow cytometric immunophenotyping of the bone marrow specimen gated on CD19 vs. forward scatter. The small B-lymphoid cells are in red while the large B-lymphoid cells are blue. C) CD5 vs. CD23 plot: the small B-lymphoid cells (red) show a coexpression of CD5 and CD23 while the large lymphoid cells (blue) are positive for CD5 at a higher intensity than small lymphoid cells and are negative for CD23. D) CD10 vs. CD20 plot: the small B-lymphoid cells (red) show dim CD20 and are negative for CD10. Large lymphoid cells (blue) are dimly positive for CD20 and show CD10 coexpression. E) CD22 vs. FMC7 plot: the small B-lymphoid cells (red) show dim CD22 and are negative for FMC7. Large lymphoid cells (blue) are positive for CD22 at a higher intensity than small lymphoid cells and are dimly positive for FMC7. F) Kappa vs. lambda plot: both the small lymphoid cells and large lymphoid cells show kappa light chain restriction.

A 57-year-old male presented with history of fever for 3 months and the presence of mediastinal lymphadenopathy, hepatosplenomegaly, and raised serum lactate dehydrogenase levels. The complete blood count revealed hemoglobin of 9.1 g/dL, total leukocyte count of 9600/µL, and platelet count of 16,000/µL. A peripheral smear revealed a leukoerythroblastic blood picture with 30 nRBCs/100 WBCs, left shift, and 6% abnormal lymphoid cells (neutrophils: 43%, lymphocytes: 42%, monocytes: 8%, myelocytes: 3%, and metamyelocytes: 4%). The bone marrow aspirate showed 10%-15% abnormal lymphoid cells, which were 3-5 times the size of a small mature lymphocyte with a moderate amount of deep blue cytoplasm, round nuclei with irregularity of membranes in some, and coarsely clumped chromatin (Figure 1A).

**Figure 2:**
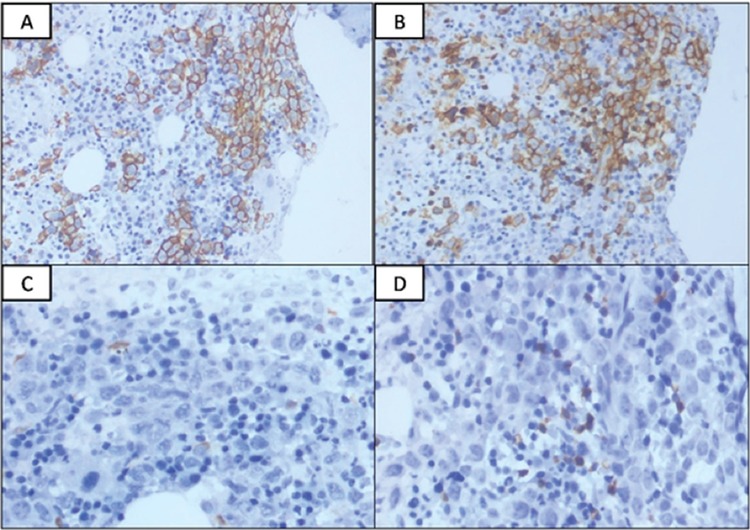
CD20 immunostaining highlights the large B cells and some small lymphoid cells are also positive (200^x^). B) Both large and small lymphoid cells show CD5 expression (200^x^). C) Cyclin D1 is negative in both small and large lymphoid cells (400^x^). D) The small lymphoid cells present interstitially are positive for CD23 (400^x^).

Immunophenotyping of the bone marrow specimen revealed two distinct populations of cells: CD19^+^ small-sized lymphocytes (red) and CD19^+^ large lymphoid cells (Figure 1B). The small cells (red) showed the CLL immunophenotype with kappa light chain restriction and CD19^+^ large cells (blue), which also showed surface kappa restriction but were CD5^+^, CD10^+^, and CD23- (Figures 1C,1D,1E,1F). The monoclonal B-cell count was 653/µL and hence the small cells represent MBL with the CLL phenotype. The large B cells suggested marrow infiltration by DLBCL. The bone marrow biopsy showed abnormal large lymphoid cells and interstitial infiltrate of small lymphocytes. Immunohistochemistry revealed that large cells were positive for CD20 (Figure 2A) and CD5 (Figure 2B) and negative for CD3, CD23, and CD10. The lymphoid cells were negative for cyclin D1 (Figure 2C). The Ki-67 staining of large lymphoid cells showed a high proliferative index (~90%). The small lymphoid cells present interstitially showed positivity for CD20, CD23, and CD5 (Figures 2A, 2B, and 2D). The immunohistochemical marker p53 seen in transformed CLL [6] was negative. Therefore, we concluded that there was incidentally detected MBL in this patient who otherwise had CD5^+^ DLBCL.

This patient received 2 doses of injection rituximab without any additional chemotherapy as he had hepatic dysfunction and poor ejection fraction. He had an aggressive disease course and succumbed within 10 days.

This report describes a rare situation where MBL was detected concomitantly with a CD5^+^ DLBCL. Gene mutation studies are necessary to differentiate these two entities. This case also calls for a consensus on reporting such cases.
